# Platelet-rich fibrin in dental extraction sockets of patients with planned or previous head and neck radiotherapy: a systematic review

**DOI:** 10.1038/s41405-026-00491-4

**Published:** 2026-11-13

**Authors:** Jaymit Patel, Clare Granger, Eleni Besi, Anastasios Kanatas

**Affiliations:** 1 Department of Restorative Dentistry, Leeds Teaching Hospitals Trust, Leeds, UK; 2 Leeds Community Healthcare NHS Trust, Leeds, UK; 3 Department of Oral Surgery & Oral and Maxillofacial Surgery, Queen Mary University of London & Barts Health Trust, London, UK; 4 Department of Oral and Maxillofacial Surgery, Leeds Teaching Hospitals Trust, Leeds, UK

**Keywords:** Oral cancer, Maxillofacial surgery

## Abstract

**Background:**

Patients with head and neck cancer (HNC) planned for radiotherapy (RT) frequently require pre-treatment dental extractions. Osteoradionecrosis (ORN) is a serious late complication of RT. Maximising the extent of socket healing before RT commences is critical to the prevention of ORN. Platelet-rich fibrin (PRF) concentrates autologous growth factors and may accelerate socket healing.

**Purpose:**

To analyse the literature and identify whether PRF supports earlier healing or reduced incidence of ORN.

**Study design, setting, sample:**

This systematic review evaluates clinical evidence for PRF in dental extraction sockets in patients with planned or prior head and neck RT. PubMed/MEDLINE, Embase, Cochrane Library, Web of Science, and the HaNDLE-on-QoL database were searched from database inception to December 2025.

**Predictor/Exposure/Independent variable:**

Studies of any design evaluating PRF in extraction sockets of patients with HNC receiving or planned for RT were eligible.

**Main outcome variable(s):**

The primary outcome variables were time to healing and incidence of ORN.

**Covariates:**

Covariates were assessed based on reporting in individual studies.

**Analysis:**

Studies were assessed for methodological robustness, including the PRISMA 2020 framework. Risk of bias was assessed using the Cochrane Risk of Bias (RoB) 2.0 tool.

**Results:**

Two primary studies, both randomised controlled trials (RCTs), met the inclusion criteria for the qualitative synthesis; one systematic review with meta-analysis was retained separately as contextual evidence. Neither RCT demonstrated a statistically significant benefit over conventional socket management. The single systematic review identified only one eligible PRF study (*n* = 11), precluding meta-analysis. No studies were conducted in the pre-RT setting.

**Conclusions:**

Current evidence is insufficient to recommend the routine use of PRF for ORN prevention in this population. Critically, both RCTs were substantially underpowered and at high risk of type II error. Both studied only post-irradiated patients and administered antibiotics to every participant in both arms, which is likely to have suppressed the baseline ORN event rate. No pre-RT evidence exists, despite this representing the most clinically and biologically relevant scenario. Adequately powered, multicentre RCTs in the pre-RT setting are required.

**Clinical trial number:**

Not applicable.

## Introduction

Head and neck cancer (HNC) encompasses malignancies of the oral cavity, oropharynx, larynx, hypopharynx, nasopharynx, and salivary glands and represents the seventh most common cancer globally [1]. Radiotherapy (RT) remains a common treatment modality for HNC. Despite advances in delivery technique, the proximity of the maxilla and mandible to RT target volumes can result in significant radiation-related oral morbidity [2–4]. Osteoradionecrosis (ORN) of the jaw is a serious late complication [5]. According to the Delanian–Lefaix fibroatrophic model [6, 7], RT-induced free radical generation triggers endothelial apoptosis and fibroblast-to-myofibroblast transformation. This results in a hypoxic, hypovascular, hypocellular tissue environment in which bone death exceeds repair capacity. Reported ORN incidence following post-RT dental extraction is approximately 10% [8], with the mandibular posterior region at doses exceeding 50–60 Gy at highest risk [8–10].

Patients diagnosed with HNC should undergo a pre-treatment restorative dental assessment to identify teeth of poor prognosis that require extraction before RT commences [11]. Extractions performed pre-RT carry lower ORN risk than those performed post-RT [12]. Current guidance recommends a minimum healing interval of 14–21 days between extraction and RT commencement [11]. Incomplete socket epithelialisation at RT commencement is an independent predictor of ORN [13, 14]; any intervention that accelerates socket healing within this narrow timeframe is therefore of clinical relevance.

Platelet-rich fibrin (PRF) is a second-generation autologous platelet concentrate introduced by Choukroun et al. [15]. Prepared by centrifugation of peripheral venous blood without anticoagulants, PRF yields a fibrin clot concentrated with platelets, leucocytes, and growth factors including vascular endothelial growth factor (VEGF), platelet-derived growth factor (PDGF), transforming growth factor-β (TGF-β), and insulin-like growth factor-1 (IGF-1). A more recent variant of these included concentrated growth factors (CGF). These are released progressively as the fibrin matrix degrades, providing a sustained angiogenic and osteogenic stimulus [16]. In non-irradiated extraction sockets, PRF significantly reduces postoperative pain and accelerates soft tissue healing compared to conventional management [17, 18], suggesting a plausible mechanism for benefit in the pre-RT context.

The objective of this systematic review is to evaluate the clinical evidence for PRF placement in dental extraction sockets for patients with planned or prior head and neck RT, to critically appraise the methodology of available evidence, and to identify future research priorities.

Research question: Does PRF, when placed in extraction sockets, improve healing outcomes or reduce the risk for ORN in irradiated patients?

## Methods

This review was conducted in accordance with the PRISMA 2020 statement (the completed PRISMA 2020 checklist is provided as Supplementary File 1). No protocol registration was completed prior to research initiation. The review protocol was not prospectively registered with PROSPERO or another registry; the rationale for, and implications of, the absence of prospective registration are addressed in the Limitations.

### Search strategy

Electronic searches were conducted in PubMed/MEDLINE, Embase, Cochrane Central Register of Controlled Trials, Web of Science, and the HaNDLE-on-QoL database (a dedicated repository of head and neck cancer quality-of-life literature) [19] from database inception to 31 December 2025. Searches were first run on 05/03/2026 and re-run on 29/05/2026; no additional eligible records were identified on re-running. No language restrictions were applied. The research was reviewed extensively to iteratively develop a research strategy. MeSH terms and free-text keywords included: ‘platelet-rich fibrin’, ‘PRF’, ‘L-PRF’, ‘A-PRF’, ‘CGF’, ‘platelet concentrate’, ‘dental extraction’, ‘tooth extraction’, ‘alveolar socket’, ‘radiotherapy’, ‘radiation therapy’, ‘head and neck cancer’, ‘osteoradionecrosis’, ‘ORN’, and ‘socket healing’. Within each concept block, terms were combined using the Boolean operator OR, and the three blocks were combined using AND, following the structure: (platelet concentrate terms) AND (dental extraction or socket terms) AND (radiotherapy, head and neck cancer or osteoradionecrosis terms). Database-specific controlled vocabulary (MeSH in PubMed/MEDLINE, Emtree in Embase) was mapped to each block and supplemented with free-text terms searched in title and abstract fields, with truncation applied where supported. Reference lists of included studies and relevant systematic reviews were hand-searched. Grey literature and conference abstracts were not searched, in order to restrict the synthesis to peer-reviewed and methodologically appraisable data; the implications of this for a niche evidence base are considered in the Limitations.

### Eligibility criteria

The PICO framework is presented in Table 1. Eligibility criteria are detailed in Table 2. Studies reporting PRF use in extraction sockets of patients with confirmed HNC where radiotherapy was planned or already provided were eligible. Eligible study designs were restricted to primary clinical studies (randomised and non-randomised controlled trials, cohort, and case-control studies); previously published systematic reviews and meta-analyses were not included in the primary synthesis but were used for reference-list screening and as contextual evidence. Studies were required to report at least one pre-specified outcome. Animal studies, in vitro research, studies in non-oncological patients, case reports/series, and conference abstracts were excluded.

**Table 1:** PICO Framework.

Component	Definition
Population	Adults with confirmed head and neck cancer (HNC) requiring dental extraction(s) where radiotherapy is planned or previously provided.
Intervention	Any PRF variant (L-PRF, A-PRF, T-PRF, i-PRF, CGF) placed within the dental extraction socket at the time of extraction.
Comparison	Conventional socket management without PRF: primary closure, blood clot, suturing, antibiotic prophylaxis alone, or no active intervention.
Outcomes	Primary: incidence of osteoradionecrosis (ORN). Secondary: socket healing, postoperative pain, infection, time to epithelialisation, quality of life.

**Table 2:** Eligibility criteria.

**Inclusion criteria**	
Study population	Adults (≥18 years) with confirmed head and neck cancer (HNC) requiring dental extraction(s) where radiotherapy is planned or previously provided (pre-, peri-, or post-radiotherapy).
Intervention	Any PRF variant (L-PRF, A-PRF, T-PRF, i-PRF, CGF) placed in a dental extraction socket.
Comparator	Conventional management (with or without antibiotics, primary closure, or suturing).
Outcomes	≥1 of: ORN incidence; socket healing; postoperative pain; infection; quality of life (QoL); time to epithelialisation.
Study design	Randomised controlled trials, non-randomised controlled trials, cohort studies, and case-control studies. Systematic reviews and meta-analyses were used for reference checking and contextual discussion only.
Publication	Full text peer-reviewed publications in any language.
**Exclusion criteria**	
Study type	Case reports/series, animal studies, in vitro studies, and ex vivo models.
Population	Patients without HNC or without RT exposure/plan; PRF studies outside the extraction socket context.
Disease context	Studies focused exclusively on osteonecrosis (including medication-related) without exposure to radiotherapy.
Publication type	Conference abstracts, editorials, unpublished data, dissertations, and grey literature.
Data quality	Duplicate data from the same cohort.

### Data extraction and quality assessment

**Fig. 1:**
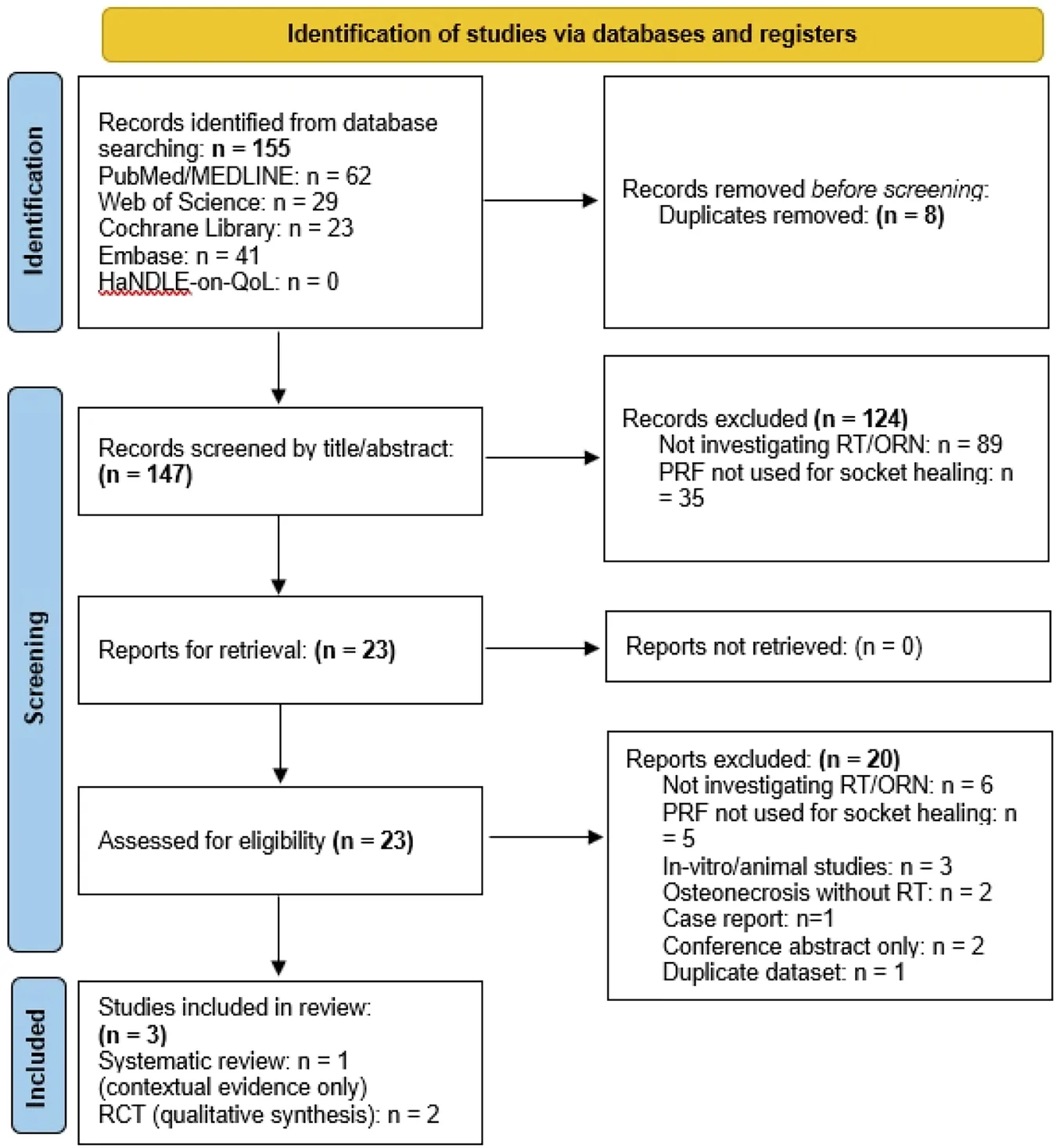
PRISMA flow diagram. RT radiotherapy, ORN osteoradionecrosis, PRF platelet-rich fibrin. The systematic review did not meet the eligibility criteria for a primary study and was retained solely for context.

**Fig. 2:**
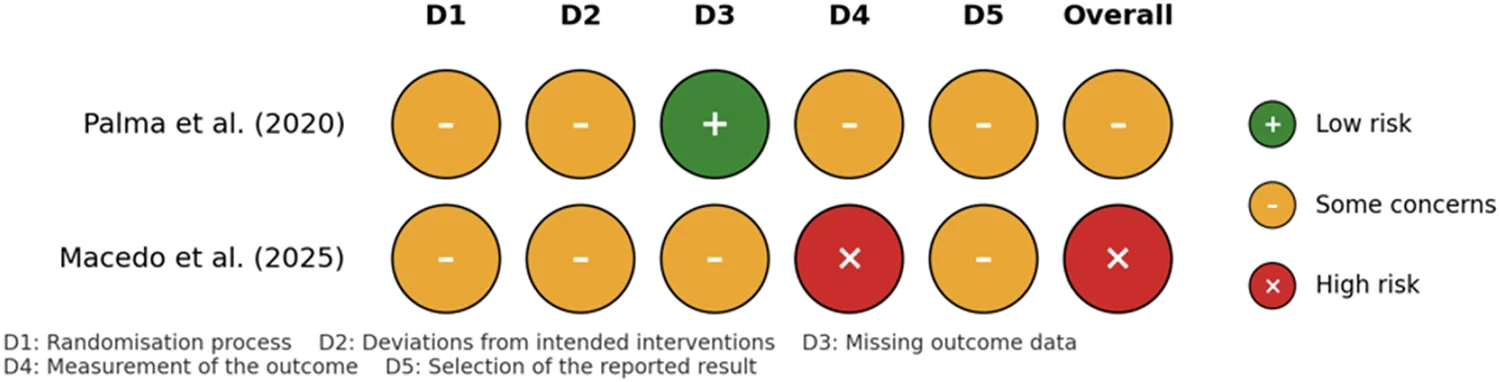
Risk-of-bias summary (traffic-light plot) for the two included randomised controlled trials, assessed with the Cochrane RoB 2.0 tool across the five domains (D1–D5) and overall.

Two reviewers independently screened titles and abstracts using the Rayyan platform [20] with blinding, followed by full-text review. Discrepancies were resolved by consensus between the two reviewers. A third author was available to facilitate discussion if required. Data extracted included: study design, year, country, sample size, RT dose and modality, PRF variant and preparation protocol, comparator, co-interventions, follow-up duration, and outcomes. For each study the unit of randomisation and the unit of statistical analysis were recorded separately, together with the number of extraction sockets contributed per participant and whether the analysis made any allowance for clustering of multiple sockets within the same patient. Risk of bias in RCTs was assessed using the Cochrane Risk of Bias (RoB) 2.0 tool [21]. GRADE certainty ratings were assigned to pooled evidence where available from included systematic reviews [22]. The study selection process, including the numbers of records identified, screened, assessed for eligibility, and excluded with reasons, is summarised in the PRISMA 2020 flow diagram (Fig. 1). A risk-of-bias summary (traffic-light plot) for the two included RCTs, generated with the RoB 2.0 tool, is provided in Fig. 2. The systematic review retained as contextual evidence (Quah et al.) was not formally appraised using a dedicated critical-appraisal instrument (e.g. AMSTAR-2), as it did not form part of the primary synthesis; this is acknowledged as a limitation.

### Ethical approval

This study did not involve human participants and represents a review of existing publications. Consequently, a human ethics application is not applicable. The accepted standards as outlined by the Declaration of Helsinki were upheld. Ethical approval was not required.

## Results

### Study selection

The search identified 155 records (PubMed/MEDLINE *n* = 62, Embase *n* = 41, Web of Science *n* = 29, Cochrane Central Register of Controlled Trials *n* = 23, HaNDLE-on-QoL *n* = 0); eight duplicates were removed. After screening titles and abstracts (*n* = 147), 23 full texts were assessed for eligibility. Two primary studies, both RCTs, were included in the qualitative synthesis (Fig. 1); one systematic review with meta-analysis (Quah et al.) was retained separately as contextual evidence and was not included in the primary synthesis. The two categories are reported separately throughout and are distinguished in Fig. 1: only the two RCTs met the eligibility criteria and contributed to the qualitative synthesis, whereas the systematic review did not meet those criteria as a primary study and was retained solely to contextualise the findings. No study examined PRF use specifically in the pre-RT extraction socket context.

### Included studies

Palma et al. [22] conducted an RCT in 23 irradiated HNC patients randomised to L-PRF (*n* = 11) or conventional management controls (*n* = 12), all of whom had received conventionally fractionated three-dimensional conformal radiotherapy to a total dose of 60–70 Gy. Eligibility was restricted to patients requiring a single tooth extraction, so each participant contributed one socket to the study. The patient was therefore both the unit of randomisation and the unit of analysis. All participants received perioperative antibiotics (clindamycin 300 mg eight-hourly for 10 days, commencing three days pre-operatively) alongside an atraumatic surgical protocol. L-PRF was prepared from a 20 mL peripheral venous sample collected into two vacuum tubes containing clot activator and centrifuged at approximately 400 *g* for 12 minutes (Daiki DT-4000, Ionlab, Brazil); two clots were produced per patient, one chopped to fill the socket and one compressed to cover it, retained with an X suture. No case of ORN, and no other postoperative complication, occurred in either arm within the 180-day follow-up (0/11 versus 0/12). A single participant, in the L-PRF arm, reported postoperative pain (visual analogue score 2 on day 3), with no significant difference between arms at day 3 or day 7 (*p* = 0.307). The trial used a convenience sample, and no a priori sample size calculation was reported.

Macedo et al. [23] reported a double-blind, split-mouth RCT in 30 post-irradiated HNC patients (radiation dose > 50 Gy; most commonly 70.2 Gy) contributing 134 extraction sockets. The socket, rather than the patient, was the unit of randomisation. Sockets were allocated to A-PRF (*n* = 67) or clot maintenance alone (*n* = 67), with each participant contributing sockets to both arms (a mean of approximately 4.5 sockets per patient). The authors state that, for statistical purposes, each post-exodontia socket was considered an individual case. Clustering was accounted for only in part: a linear mixed model was applied to the continuous healing index. Wound closure, bone exposure, ORN and oedema were compared using Fisher’s exact test, which treats sockets as independent. A-PRF was prepared from two additive-free 9 mL glass tubes (18 mL total) centrifuged at 1300 rpm (208 g) for 14 minutes (Duo Quattro, Process for PRF, Nice, France), with a single clot placed per socket. All patients received amoxicillin 500 mg eight-hourly from three days pre-operatively and for seven days postoperatively. The sample size was calculated for degree of complete epithelialisation rather than for ORN, and 30 of the 32 patients indicated by that calculation were enrolled. Clinical evaluation was undertaken at 7, 14, 30, 60, 90 and 120 days. No case of ORN was recorded in either arm at 90 or 120 days (0/67 versus 0/67), and median pain scores were zero in both arms at every time point. Complete wound closure was, however, more frequent in the control arm than in the A-PRF arm at 7 days (58/67, 86.5% versus 49/67, 73.1%; unadjusted risk difference −13.4%, 95% CI−26.6% to 0.2%) and at 14 days (57/67, 85.0% versus 47/67, 70.1%; unadjusted risk difference −14.9%, 95% CI−28.5% to −0.7%), reported by the authors as statistically significant.

As contextual evidence, Quah et al. [24] conducted a systematic review and meta-analysis of five adjunctive modalities in post-radiotherapy extraction sites across 29 studies (*n* = 1520 patients). Only one PRF study was considered eligible. This study recruited 11 participants, with an ORN prevalence of 0%. Pooled ORN rates for the other groups were: hyperbaric oxygen (HBO) 4.6%, pentoxifylline-tocopherol (PENTO) 3.4%, and antibiotics 3.8%, compared with 17.6% in untreated controls. GRADE certainty of evidence for PRF was rated ‘very low’.

A summary of the included studies is presented in Table 3.

**Table 3:** Summary of Included Studies.

Author (year)	Design	*n* (patients / sockets); unit of randomisation	PRF type and preparation protocol	RT timing	Follow-up	Primary outcome	Key result
Palma et al. [23]	RCT	23 patients, 1 socket each; patient	L-PRF. 20 mL venous blood into 2 clot-activator tubes; ≈400 g for 12 min (Daiki DT-4000, Ionlab, Brazil); 2 clots per patient — 1 chopped to fill the socket, 1 compressed to cover it. Antibiotic: clindamycin 300 mg 8-hourly, 10 days.	Post-RT	180 days	ORN incidence	0% (*n* = 11) vs 0% (n = 12) ORN — no case in either arm at 180 days. RD 0% (95% CI−24.2% to 25.9%). No difference in pain at day 3 or 7 (*p* = 0.307).
Macedo et al. [24]	RCT	30 patients/ 134 sockets (split-mouth); socket	A-PRF. 18 mL venous blood into 2 × 9 mL additive-free glass tubes; 1300 rpm (208 *g*) for 14 min (Duo Quattro, Process for PRF, Nice, France); 1 clot per socket. Antibiotic: amoxicillin 500 mg 8-hourly, 3 days pre-operatively and 7 days after.	Post-RT	120 days	Pain & healing	0% (*n* = 67) vs 0% (*n* = 67) ORN — no cases in either arm at 120 days; unadjusted RD 0% (95% CI−5.4% to 5.4%). Median pain 0 in both arms at all time points. Complete wound closure was lower with A-PRF at 7 days (73.1% vs 86.5%) and 14 days (70.1% vs 85.0%), reported by the authors as *p* < 0.05 in favour of the control.
Quah et al. [25]	SR/MA	1520 total, with 11 participants in the PRF study	A-PRF (1 study)	Post-RT	Variable	ORN incidence	0% ORN (*n* = 11)

*RCT* randomised controlled trial, *RT* radiotherapy, *ORN* osteoradionecrosis, *SR/MA* systematic review and meta-analysis, *RD* risk difference, *CI* confidence interval. Risk differences and confidence intervals were calculated for this review from published event counts (Newcombe hybrid score method) and are unadjusted for clustering of sockets within patients.

## Discussion

The existing evidence is structurally unable to answer the clinical question in its current form. The absence of a statistically significant finding in both RCTs should not be interpreted as evidence that PRF is ineffective; rather, it indicates that the trials lacked the power to detect a clinically meaningful effect had one been present. Assuming the accepted prevalence of ORN [8, 10, 25, 26] and a hypothesised 50% relative risk reduction [18], a two-arm RCT requires approximately 425 patients per arm (assuming a control-arm ORN incidence of 10%, two-sided α = 0.05 and 80% power) to be adequately powered. Palma et al. [22] enrolled 11 patients in the PRF arm; and Macedo et al. [23] enrolled 67 sockets (not independent patients). Both trials were therefore operating at a statistical power of approximately 5–10% for this outcome. Failure to detect a benefit under such conditions places these trials at high risk of a type II error. Neither trial was designed to test ORN as an outcome (due to the method in which sample size was calculated). Palma et al. used a convenience sample with no a priori power calculation, and Macedo et al. calculated their sample size for degree of epithelialisation rather than for ORN. The assumed 50% relative risk reduction is, however, an optimistic extrapolation: it is derived from a meta-analysis of PRF in soft-tissue healing and recovery after third molar surgery in non-irradiated patients [18], and no equivalent effect has been demonstrated for ORN, which is a bone pathology mechanistically distinct from soft-tissue socket healing. The required sample size is highly sensitive to this assumption: holding the baseline ORN incidence at 10% (two-sided α = 0.05, 80% power), a 50% relative risk reduction requires approximately 425 patients per arm, a 40% reduction approximately 715 per arm, and a 30% reduction approximately 1350 per arm. The estimate of ‘5–10% power’ for the existing trials should accordingly be read as an order-of-magnitude figure under these stated assumptions rather than a precise value.

While the results provide valuable insights, they should be tempered by several inherent methodological constraints. Osteoradionecrosis (ORN) takes significant time to develop. Consequently, there is a natural scarcity of published research. The small number of available papers limits the ability to generalise these findings across broader populations.

The most clinically relevant finding of this review is the complete absence of studies in the pre-RT context. In the “pre-RT socket”, tissue vascularity is intact and the wound healing cascade is unimpaired. The biological case for PRF is stronger here than in post-RT tissue, where Delanian and Lefaix [7] demonstrated that RT-induced fibroatrophy produces progressive endothelial dysfunction. Haimovitz-Friedman et al. [27] showed that ionising radiation alters growth factor gene expression in endothelial cells in a dose- and time-dependent manner, including the PDGF and bFGF signalling axes through which PRF exerts its principal angiogenic effects. In the post-RT microenvironment, therefore, the cellular mechanism on which PRF depends may itself be compromised. As such, the two existing RCTs have evaluated PRF in the post-RT setting, where it is least likely to impart a biological benefit.

Incomplete socket epithelialisation at RT commencement is associated with an increased risk of ORN [13]. Systematic review evidence demonstrates that PRF significantly accelerates soft tissue closure in non-irradiated extraction sockets at 7- and 14-days post-extraction [17, 18]. If this benefit is reproducible in the pre-RT population, even a modest reduction in the healing interval could have a substantial clinical impact on the risk of ORN. This hypothesis is biologically coherent, clinically important, and untested in clinical trials. A distinction should be drawn between biological plausibility and demonstrated clinical effectiveness. No clinical study has evaluated PRF in a pre-RT extraction socket, and no claim about its effectiveness in that setting can currently be supported by direct evidence.

Three interacting methodological problems undermine the available RCTs beyond sample size alone.

First, universal antibiotic co-intervention constrains interpretability, though not through confounding in the conventional sense. Quah et al. [25] reported that antibiotics alone reduce ORN from 17.6% to 3.8%: a 78% relative risk reduction. Because antibiotics were administered to every participant in both arms of both trials, they cannot have generated a systematic difference between the groups and are not, strictly, a confounder of the treatment comparison. Their influence is instead on the baseline event rate. By suppressing the incidence of ORN in the control condition towards zero, they leave little residual risk for PRF to reduce, so that any additional effect of PRF becomes correspondingly more difficult to detect at the sample sizes studied. The observed outcome (no case of ORN in either arm of either trial) is consistent with this interpretation. A further areas for consideration is L-PRF has itself been shown to act as a local reservoir for antibiotic release, so the two interventions may not be wholly independent of one another [28].

Second, the follow-up duration was inadequate to assess most ORN events. Balermpas et al. [29] reported a median time to ORN onset of 12.8 months in intensity-modulated radiotherapy (IMRT)-treated cohorts, with substantial proportions presenting beyond 24 months. Both RCTs used a 120–180-day follow-up. Their ORN event rates would therefore be systematically underestimated, further increasing the risk of false negatives.

Third, PRF product heterogeneity invalidates cross-study comparisons. Dohan Ehrenfest et al. [30] established that centrifugation conditions are the primary determinant of PRF composition, with up to five-fold variation in PDGF-AB and TGF-β1 content between L-PRF (400 g) and A-PRF (200 g) protocols [31]. Both RCTs did in fact report relative centrifugal force: approximately 400 g for 12 minutes in Palma et al. and 208 g (1300 rpm) for 14 minutes in Macedo et al. (the standardised parameter recommended by Ghanaati et al. [32]). The difficulty is therefore not one of reporting but of comparability. The two protocols differ approximately two-fold in RCF and also in spin duration, tube type, blood volume and the number of clots placed per socket, and were performed on different centrifuges. On the data of Dohan Ehrenfest et al. and Miron et al., differences of this magnitude would be expected to yield materially different growth factor content, so the two trials cannot be assumed to have tested the same biological product. Pooling them without accounting for this heterogeneity would be methodologically questionable, and future trials should report RCF, spin time, rotor geometry, blood volume and clot number in full.

The 0% ORN rate in the single PRF study identified by Quah et al. [25] compares numerically favourably with pooled rates for HBO (4.6%), PENTO (3.4%), and antibiotics (3.8%). However, this result is based on only 11 patients and carries a GRADE certainty of ‘very low’. It would therefore be inappropriate to use this figure to position PRF above better-studied modalities.

A randomised controlled clinical trial investigating the effectiveness of PRF in reducing the prevalence of ORN in pre-RT extraction sites would be of clear value, although feasible only as a multicentre collaboration given the sample size required. Minimum design requirements are proposed in Appendix 1. Key features include: prospective enrolment of pre-RT patients; a sample size of at least 850 (approximately 425 per arm) based on a 10% baseline ORN rate and a hypothesised 50% relative risk reduction; standardised A-PRF preparation protocol (RCF-reported [33]); primary endpoint of ORN at 24 months; and prospective trial registration with a pre-specified statistical analysis plan.

The absence of evidence of benefit is not evidence of harm, but neither does it constitute grounds for recommending use. PRF is autologous, has no reported systemic adverse effects and imposes a modest procedural burden; set against this, the only comparative data available in irradiated patients show no benefit, and in one trial showed less complete wound closure in the PRF arm at 7 and 14 days. Given the quantity and quality of the available evidence, PRF cannot be recommended for routine use and any use should be regarded as investigational. Where a clinician nonetheless elects to use it, it should be strictly as an adjunct to, and never a substitute for, established preventive measures (atraumatic technique, primary closure, adjunctive antibiotics, and adequate healing time before RT). It has been suggested that use might reasonably be prioritised in patients at highest risk, and it should be emphasised that this proposition rests on biological reasoning alone. It is also in precisely these highest-risk patients that an ineffective adjunct carries the greatest opportunity cost, should it displace or delay measures of established value. Informed consent should state explicitly that no clinical trial evidence of benefit exists in this setting.

### Limitations

This review has several limitations. First, the review protocol was not prospectively registered, for example with PROSPERO. Prospective registration is increasingly expected for systematic reviews, and its absence raises the possibility of publication or selective-reporting bias and reduces transparency regarding any deviation from a pre-specified plan. Registration was not undertaken because the review addressed a deliberately narrow question for which the eligible primary literature was known to be very small; to mitigate the associated risks, eligibility criteria were defined a priori, study screening and data extraction were performed independently in duplicate, and reporting follows the PRISMA 2020 statement (Supplementary File 1).

Second, grey literature and conference abstracts were not searched. In a niche field, this may have excluded relevant pilot data or ongoing work; notably, at least one trial of PRF applied before radiotherapy is registered and ongoing (ClinicalTrials.gov NCT06406530), indicating that direct evidence for the pre-RT setting may emerge in the coming years.

Third, only two small primary studies were eligible, both performed after radiotherapy; this limits the generalisability of any synthesis and precluded meta-analysis. The systematic review by Quah et al. was used only as contextual evidence and was not formally appraised with a critical-appraisal instrument such as AMSTAR-2, and this should be borne in mind when interpreting the pooled figures it reports. Finally, heterogeneity in PRF preparation, comparator regimens, universal antibiotic co-intervention and follow-up duration across the available studies constrains direct comparison and represents an important potential source of bias, as discussed above.

## Conclusions

PRF is biologically plausible as an adjunct in pre-RT dental extraction sockets, but biological plausibility is not clinical effectiveness, and no direct evidence of benefit exists in that setting. The existing clinical evidence, derived solely from post-irradiated patients in two substantially underpowered RCTs at high risk of type II error, is insufficient either to support or to refute its routine use. The pre-RT setting remains uninvestigated, and no direct clinical evidence currently exists for this setting. Well-designed, adequately powered, multicentre RCTs enrolling pre-RT HNC patients are a research priority. Until such evidence is available, PRF should be considered investigational, should not be presented to patients or colleagues as an established means of reducing ORN risk, and where used should be used only as an adjunct to established preventive strategies, with its application documented so as to contribute to the real-world evidence base.

## Supplementary information

Supplementary File 1

## Appendix

Appendix 1 Proposed minimum design criteria for a definitive RCT

**Table:**

Design element	Specification
Population	Adults with HNC planned for head and neck RT requiring ≥1 pre-RT dental extraction; no prior jaw RT; enrolled at pre-treatment dental assessment.
Intervention	Standardised A-PRF (200 g/8 min; RCF-reported per Ghanaati et al. [27]) in all extraction sockets; confirmed by photodocumentation.
Comparator	Atraumatic extraction + primary closure + perioperative antibiotics; no PRF.
Primary outcome	ORN incidence (SICMF/SIPMO staging [28]) at 24 months post-RT commencement; adjudicated by blinded radiologist and clinician.
Sample size	*n* ≥ 1000 (approximately 500/arm) assuming 10% baseline ORN, 50% relative risk reduction, 80% power, α = 0.05, 15% dropout.
Secondary outcomes	Time to socket epithelialisation (blinded photography); socket bone fill at 3 months (CBCT); pain (VAS d1, d3, d7); OHIP-14 at 3, 6, 12, 24 months.
Randomisation	Central web-based; stratified by RT dose (≤50 Gy vs. >50 Gy), socket site (mandibular posterior vs other), antibiotic co-prescription, and RT modality (IMRT vs VMAT), with the planned mandibular dose-volume recorded to account for variability between modern radiotherapy techniques.
Blinding	Patient-blinded (standardised dressing). Outcome assessors blinded. Operators unblinded; interoperator reliability testing required.
Registration	Prospective ISRCTN/ClinicalTrials.gov registration; pre-specified SAP; annual DSMB review. Funnel plot assessment mandatory in any subsequent MA.

*RCF* relative centrifugal force, *CBCT* cone beam computed tomography, *VAS* visual analogue scale, *OHIP-14* Oral Health Impact Profile-14, *SAP* statistical analysis plan, *DSMB* Data Safety and Monitoring Board; MA = meta-analysis.
